# Numerical Study on Crack Propagation in Brittle Jointed Rock Mass Influenced by Fracture Water Pressure

**DOI:** 10.3390/ma8063364

**Published:** 2015-06-09

**Authors:** Yong Li, Hao Zhou, Weishen Zhu, Shucai Li, Jian Liu

**Affiliations:** 1 Geotechnical & Structural Engineering Research Center, Shandong University, Jinan 250061, Shandong, China; E-Mails: haozhouchina@gmail.com (H.Z.); zhuw@sdu.edu.cn (W.Z.); lishucai@sdu.edu.cn (S.L.); 2 School of Civil Engineering, Shandong University, Jinan 250061, Shandong, China; E-Mail: lj75@sdu.edu.cn

**Keywords:** fracture propagation, jointed rock mass, fracture water pressure, numerical simulation

## Abstract

The initiation, propagation, coalescence and failure mode of brittle jointed rock mass influenced by fissure water pressure have always been studied as a hot issue in the society of rock mechanics and engineering. In order to analyze the damage evolution process of jointed rock mass under fracture water pressure, a novel numerical model on the basis of secondary development in fast Lagrangian analysis of continua (FLAC3D) is proposed to simulate the fracture development of jointed rock mass under fracture water pressure. To validate the feasibility of this numerical model, the failure process of a numerical specimen under uniaxial compression containing pre-existing fissures is simulated and compared with the results obtained from the lab experiments, and they are found to be in good agreement. Meanwhile, the propagation of cracks, variations of stress and strain, peak strength and crack initiation principles are further analyzed. It is concluded that the fissure water has a significant reducing effect on the strength and stability of the jointed rock mass.

## 1. Introduction

To a great extent, it is the nearly ubiquitous presence of fractures that makes the mechanical behavior of rock masses different from that of most engineering materials. These fractures have a controlling influence on the mechanical behavior of rock masses, since existing fractures provide planes of weakness on which further deformation can more readily occur. Fractures also often provide the major conduits through which fluids can flow [1]. As the Chinese economy gradually grows, the Chinese government will begin to construct numerous huge engineering projects, like hydropower stations, mining, tunnels, large-scale underground caverns for energy storage, *etc.* Therefore, the related problems in jointed rock mass will be encountered in the future [2]. A series of cracking processes finally control the overall behavior of the rock, which have prompted extensive experimental studies of pre-cracked specimens of different materials, including rock-like brittle/semi-brittle materials and natural rocks: glass [3], molded gypsum [4], sand-stone-like material [5], granite [6], marble [7], *etc.* Numerous numerical methods have also been used to simulate the fracture development. These methods could be divided into two types: continuous and discontinuous numerical methods. Tang *et al.* [8] developed some numerical methods to simulate the initiation and coalescence of flaws in rock-like materials, including the finite element method (FEM), boundary element method (BEM) and displacement discontinuity method (DDM), and Tang [9] also proposed a new numerical code named RFPA2D (Rock Failure Process Analysis) to simulate the propagation and coalescence of cracks in a rock bridge area. In addition, the discrete element method (DEM) is also used to simulate the mechanical behavior of rock-like materials [10,11,12,13]. The above research was not entirely conducted under the conditions of fissure water pressure. Fang and Harrison [14,15] adopted a degradation model to simulate the brittle failure in heterogeneous rocks. Xie *et al.* [16] proposed a micromechanical analysis of damage and related inelastic deformation in saturated porous quasi-brittle materials in 2012. Then, Zhu *et al.* [17] gave a deep discussion about two dissipative processes in microcracks. Bikong *et al.* [18] proposed a micro-macro model for the time-dependent behavior of clayey rocks in 2015. Richardson *et al.* [19] presented a method for simulating quasi-static crack propagation in 2D, which combines XFEM with a simple integration technique and a very general algorithm for cutting triangulated domains. In this paper, to simulate the fissure development of jointed rock mass under fissure water pressure, we propose a novel numerical model on the basis of secondary development in Lagrangian analysis of continua (FLAC3D) [20], which is an explicit finite difference method (FDM). Finally, the numerical model is used to study the fissure development of rock specimens.

## 2. An Elastic-Brittle Constitutive Model and Hydro-Mechanical Coupling

### 2.1. An Elastic-Brittle Constitutive Model

**Figure 1:**
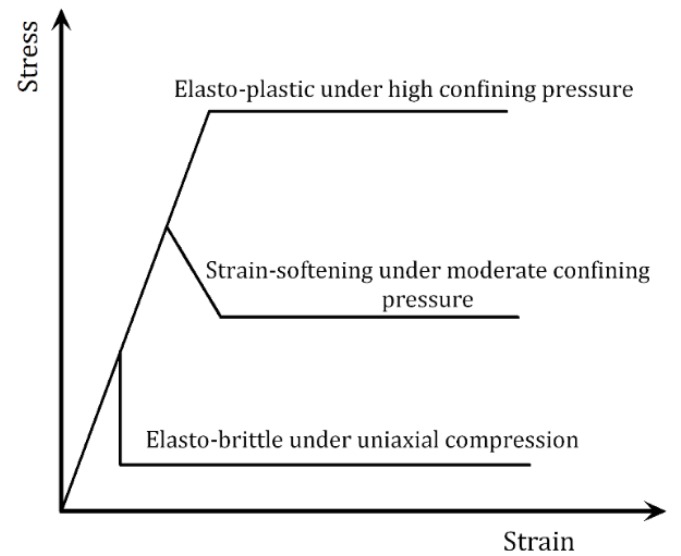
Simplified stress-strain relations of rock elements under different confining pressures within a stressed rock body.

As is known to us all, the nonlinear stress-strain relationship of brittle materials, like rock, concrete *etc.*, does not result from plastic deformations. It is caused by the initiation, propagation and coalescence of the micro-cracks in heterogeneous materials. Therefore, it is appropriate to adopt an elastic-damage model to describe the micro-mechanical properties of brittle materials. The behavior of the rock element undergoing failure, as used in the analysis of the behavior of a rock specimens [21,22], may be simplified to either elastic-brittle, elastic-strain softening (a combination of brittle and ductile) or elastic-ductile (plastic) mechanisms, as shown in Figure 1.

The above elastic-plastic model and strain-softening model could not effectively simulate the failure development of rock materials; even some microscopic problems are difficult to be solved due to the large plastic zone appearing in the crack tips. According to the curves of elastic-brittle stress-strain relations, a piecewise function could be used to express the whole process of the stress-strain relations.

As for the post-failure elements, the mechanical properties must be degraded, and the stress field must be redistributed. Consequently, tensile failure occurs, and cracks are initiated. This model could be effectively used to simulate the complex fissure development in heterogeneous materials. In this elastic-brittle damage model, the specimens under uniaxial tensile loads still have residual strength after they undergo yield strength. This model could be expressed as the following equations:

(1) $$D=\left\{ \begin{array}{l} 0,\varepsilon <\varepsilon_{t0}\\ 1-\frac{\sigma_{i}}{\varepsilon \cdot E_{0}},\varepsilon_{t0}\leq \varepsilon \leq \varepsilon_{tu}\\ 1,\varepsilon >\varepsilon_{tu} \end{array}\right.$$

(2) $$\sigma_{i}=\eta \cdot \sigma_{t}$$

where *σ_i_* is the residual strength; *ɛ_t_*_0_ is the initial damage threshold; *ɛ_tu_* is the limit of tensile strain; *ŋ* is the residual strength coefficient; *D* represents the damage variable; and *σ_t_* is the uniaxial tensile strength.

According to Mazars’ method [23], the tensile strain *ɛ* in Equation (1) could be substituted by an equivalent strain $\bar{\varepsilon }$ in three-dimensional conditions:

(3) $$D=\left\{ \begin{array}{l} 0,\bar{\varepsilon }<\varepsilon_{t0}\\ 1-\frac{\sigma_{i}}{\bar{\varepsilon }\cdot E_{0}},\varepsilon_{t0}\leq \bar{\varepsilon }\leq \varepsilon_{tu}\\ 1,\bar{\varepsilon }>\varepsilon_{tu} \end{array}\right.$$

where $\bar{\varepsilon }=\sqrt{{\left(\varepsilon_{1}\right)}^{2}+{\left(\varepsilon_{2}\right)}^{2}+{\left(\varepsilon_{3}\right)}^{2}}$.

Based on elastic damage mechanics, the stress-strain relations of the constitutive model could be described as the following equations:

(4) $$\sigma_{ij}=\left\{ \begin{array}{l} 2G\cdot \varepsilon_{ij}+\lambda \cdot \delta_{ij}\cdot \varepsilon_{kk},\bar{\varepsilon }<\varepsilon_{t0}\\ \frac{\sigma_{i}}{\bar{\varepsilon }\cdot E_{0}}(2G\cdot \varepsilon_{ij}+\lambda \cdot \delta_{ij}\cdot \varepsilon_{kk}),\varepsilon_{t0}\leq \bar{\varepsilon }\leq \varepsilon_{tu}\\ 0,\bar{\varepsilon }>\varepsilon_{tu} \end{array}\right.$$

where $G=\frac{E_{0}}{2(1+v)}$; $\lambda =\frac{E_{0}\cdot \nu }{\left(1+\nu )\cdot (1-\nu \right)}$; and $\delta_{ij}=\left\{ \begin{array}{l} 1,i=j\\ 0,i\neq j \end{array}\right.$.

The damage evolution equations of shear failure are expressed as below:

(5) $$D=\left\{ \begin{array}{l} 0,\varepsilon_{1}<\varepsilon_{c0}\\ 1-\frac{\sigma_{rc}}{\varepsilon_{1}\cdot E_{0}},\varepsilon_{1}\geq \varepsilon_{c0} \end{array}\right.$$

where *σ_rc_* is the residual strength of shear damage; and *ɛ_c_*_0_ is the strain threshold of shear damage.

Finally, when an element is experiencing shear failure, the equations of the constitutive model could be expressed as below:

(6) $$\sigma_{ij}=\left\{ \begin{array}{l} 2G\cdot \varepsilon_{ij}+\lambda \cdot \delta_{ij}\cdot \varepsilon_{kk},\varepsilon_{1}<\varepsilon_{c0}\\ \frac{\sigma_{rc}}{\varepsilon_{1}\cdot E_{0}}(2G\cdot \varepsilon_{ij}+\lambda \cdot \delta_{ij}\cdot \varepsilon_{kk}),\varepsilon_{c0}\leq \varepsilon_{1}\leq \varepsilon_{ut}\\ 0,\bar{\varepsilon }>\varepsilon_{ut} \end{array}\right.$$

### 2.2. Hydro-Mechanical Coupling of Jointed Rock Mass

The hydro-mechanical coupling of jointed rock mass is realized by the stress equilibrium equation and the continuous seepage equation. The stress equilibrium equation is usually expressed by the principle of virtual work. This means that the virtual work difference of body forces and plane forces at any time is zero:

(7) $$\int^{\text{​}}\delta \varepsilon^{T}d\sigma dV-\int^{\text{​}}\delta u^{T}dfdV-\int^{\text{​}}\delta u^{T}dtdS=0$$

where *δε* is the virtual strain; *δμ* is the virtual displacement; *t* is the plane force; and *f* is the body force.

When porous media is considered, the expression of the Biot effective stress is:

(8) $$\sigma^{\prime }=\sigma -\alpha \bar{p}$$

where *σ′* is the effective stress; *σ* is the total stress; α  is Biot’s coefficient; and $\bar{p}$ is the average stress of the fluid. Biot’s coefficient would evolve with the damage process, but it is very difficult to obtain its variation principle during the coupling process. According to the research results of Walsh [24] and Zhao [25], Biot’s coefficient is between zero and one.

The constitutive model could be expressed by the strain increment:

(9) $$\text{d}\sigma^{\prime }=D_{ep}\left(d\varepsilon -d\varepsilon_{l}\right)$$

where *D_ep_* represents an elastic-plastic matrix; and $d\varepsilon_{l}\;$ is the particle compression induced by pore flow. Here, this is calculated as the following equation:

(10) $$\text{ε}=\sigma^{\prime }\left(1-D\right)E_{0}$$

The continuous seepage equation is expressed as below based on a hypothesis of the Darcy flow:

(11) $$S_{w}\left[m^{T}-\frac{m^{T}D_{ep}}{3K_{s}}\right]\frac{d\varepsilon }{dt}-\nabla^{T}\left[k_{0}k_{r}\left(\frac{\nabla p_{w}}{p_{w}}-\text{g}\right)\right]+\left\{\zeta n+n\frac{S_{w}}{k_{w}}+S_{w}\left[\frac{1-n}{3K_{s}}-\frac{m^{T}D_{ep}m}{{\left(3K_{s}\right)}^{2}}\right]\left(S_{w}+p_{w}\zeta \right)\right\}\frac{dp_{w}}{dt}=0$$

where *S_w_* is the degree of saturation; *p_w_* is the pore water pressure; $\text{ζ}=\frac{\text{d}s_{w}}{\text{d}p_{w}}$; *k*_0_ is the initial permeability coefficient tensor; *k_r_* is the permeability coefficient; g is the gravity acceleration vector; *n* is the porosity; and *k_w_* is the bulk modulus of water. The above equations provide the theoretical fundamentals in hydro-mechanical coupling of jointed rock mass.

## 3. Implementation of the Elastic-Brittle Coupling Model in FLAC3D

**Figure 2:**
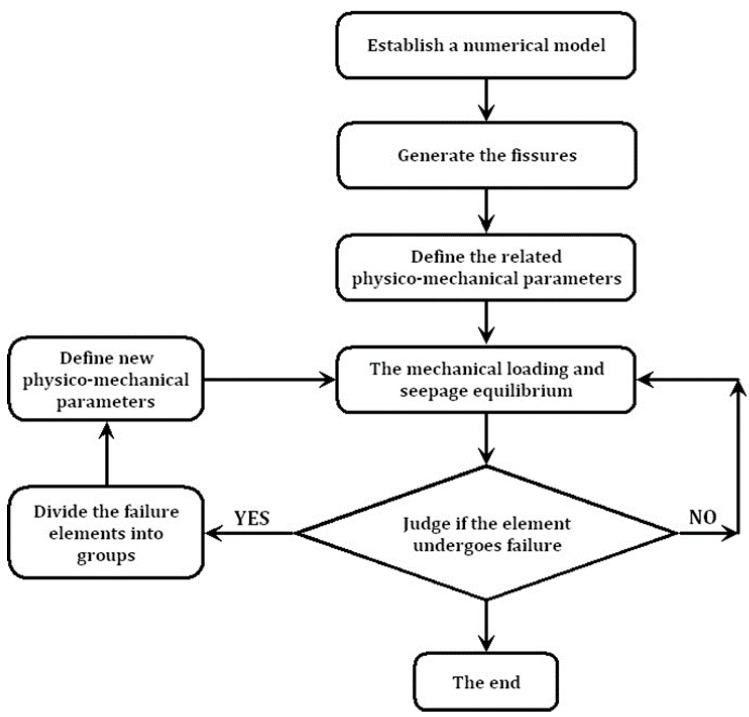
The procedure for the implementation of the elastic-brittle coupling model in Lagrangian analysis of continua (FLAC3D).

A survey of commercially available codes shows that the program fast Lagrangian analysis of continua (FLAC3D), produced by Itasca Consulting Group [20], uses an explicit finite difference scheme for the analysis of problems in engineering mechanics. FLAC3D implements an explicit time marching scheme to solve Newton’s second law to describe material deformation and embodies a number of basic constitutive models for use in the analysis of the mechanical behavior of geo-materials. Based on these, users can incorporate their own constitutive models by writing a function using a built-in programming language, which is called the FISH language. This provides an easy way to enhance the program, and hence, solve complex problems in rock mechanics and rock engineering. Thus, FLAC has been adopted for the implementation of the elastic-brittle coupling model. Figure 2 shows the procedure for the implementation of the elastic-brittle coupling model in FLAC3D.

## 4. Numerical Simulations on the Specimens Containing Precast Fissures

### 4.1. A Two-Dimensional Numerical Simulation

**Figure 3:**
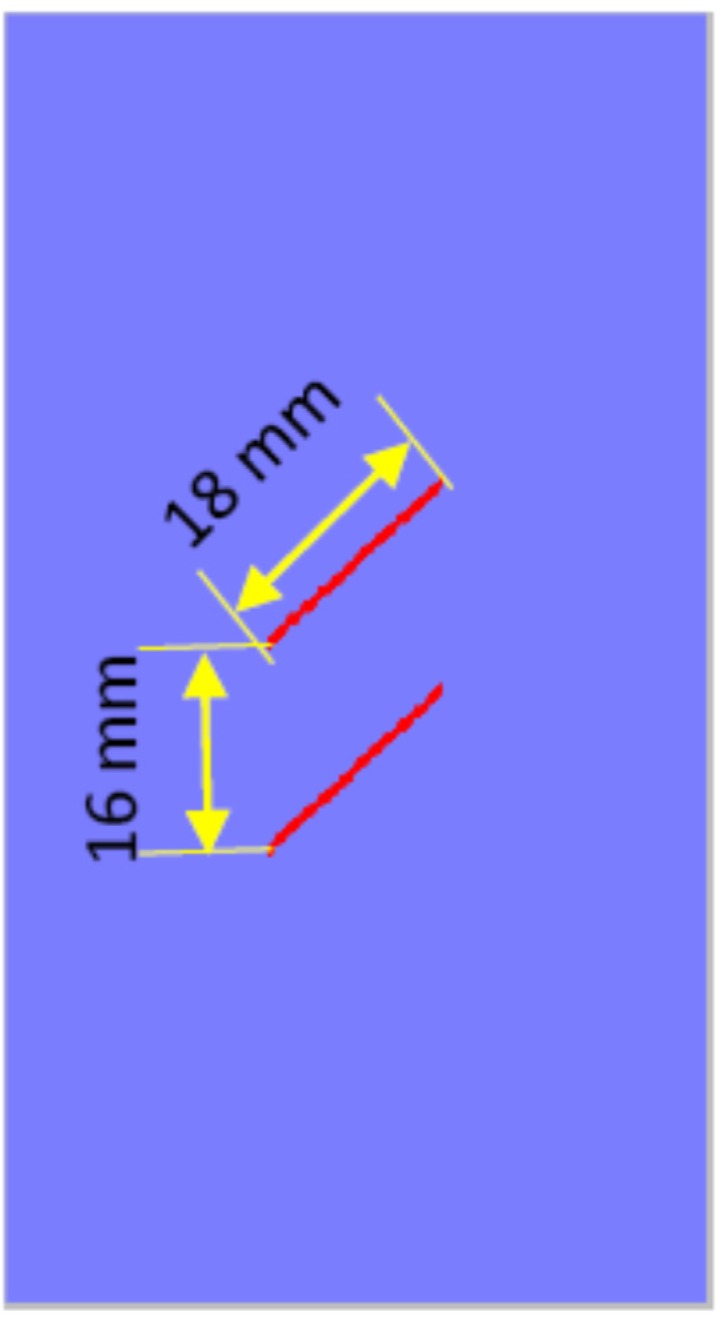
The two-dimensional numerical model.

The dimensions of the length, height and thickness in the two-dimensional numerical model shown in Figure 3 are 50 mm, 100 mm and 1 mm, respectively. This numerical model is divided into 7524 elements. It contains two types of media, the intact rock mass and precast fissures. The two parallel fissures are located in the center of the model. The vertical distance between them is 16 mm; the length of the fissure is 18 mm; the thickness is 1 mm; and the dip angle is 45°. The whole numerical model is freely meshed by hexahedral elements. The related physico-mechanical parameters are shown in Table 1.

**Table 1:** Physico-mechanical parameters of intact rock mass and precast fissure.

Rock types	Elastic modulus (GPa)	Poisson’s ratio	Tensile strength (MPa)	Cohesion (MPa)	Friction angle (°)	Dilatancy angle (°)
Intact rock mass	45.0	0.25	0.9	1.6	40	0
Precast fissure	1.5	0.35	0.5	0.8	20	0

**Figure 4:**
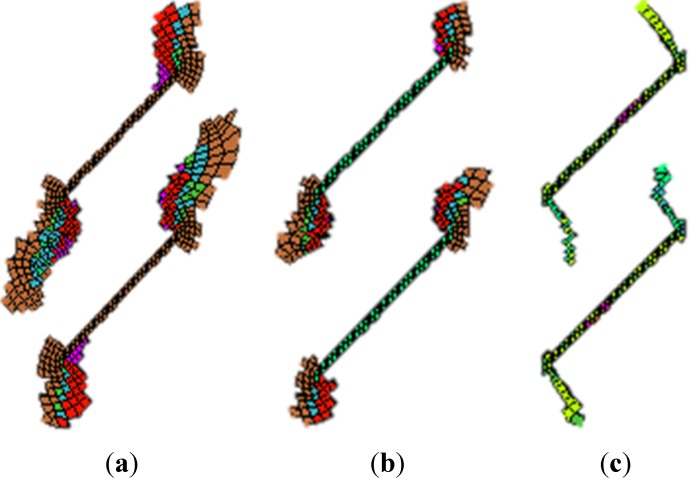
Numerical simulation results using elasto-plastic, strain softening and elastic brittle models. (**a**) Plastic zones obtained by elasto-plastic model; (**b**) Plastic zones obtained by strain-softening model; (**c**) Plastic zones obtained by elastic-brittle model.

Next, the elasto-plastic model, the strain-softening model and the elastic-brittle model are also used to simulate the fracture development under uniaxial compression. Figure 4 shows the numerical simulation results. Figure 4a is obtained by the elasto-plastic model. Although the failure occurs near the fissure tips and large-area plastic zones appear, the development of the secondary cracks could not be better observed. Figure 4b is obtained by the strain softening model. Although the plastic zone becomes smaller, it has the same difficulty as Figure 4a. Figure 4c is obtained by the elastic-brittle model. We find that the simulation results are absolutely different with those obtained by the above two models. We observe the development of secondary cracks, and no large-area plastic zones appear, which is extremely close to the results obtained in the laboratory testing specimens [26].

From the numerical results, it is concluded that the elastic-brittle model is more appropriate to simulate the fracture development of brittle geo-materials.

### 4.2. Three-Dimensional Numerical Simulations

**Figure 5:**
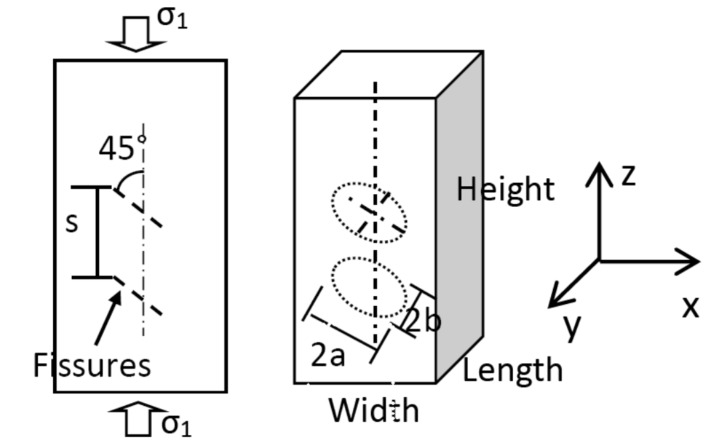
Location of double parallel fissures.

**Figure 6:**
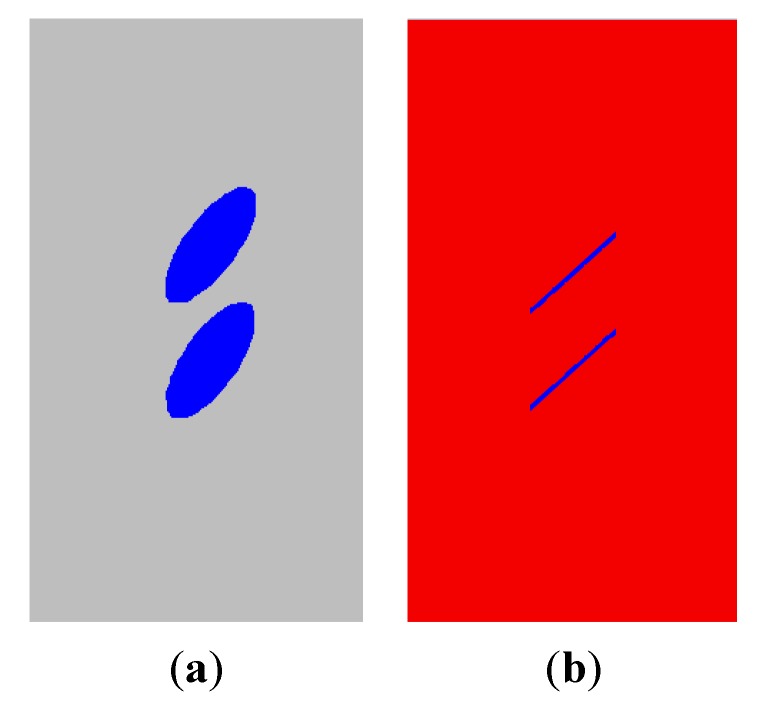
The three-dimensional model. (**a**) A side view; (**b**) A front view.

The three-dimensional numerical model adopts a cuboid model, as shown in Figure 5, and the dimensions of the length, width and height are 50 mm, 50 mm and 100 mm, respectively. Two elliptic mica sheets are used to simulate the double fissures, which is more appropriate than the metal sheets in mechanical behavior. The long axis, short axis and thickness of the elliptic fissure are 18 mm, 15 mm and 1 mm. The fissure planes have an inclination angle of 45° to the horizontal plane, and the vertical distance between them is 16 mm. The rolling constraint is fixed to the top and bottom surfaces. In order to clearly observe the fissure development, super fine meshes are generated, and the number of elements is 270,603, as shown in Figure 6. The uniaxial loading is applied on the top and bottom surfaces. The related physico-mechanical parameters are also shown in Table 1.

When the elastic brittle numerical model is used to perform the numerical simulations on the fissure development in the specimens, the following essentials should be noticed. As for the elements experiencing shear failures, their residual shear strength should be reduced to five percent of the original shear strength, and for the elements experiencing tensile failures, their tensile strength and cohesion should be reduced to only one percent of the original values. However, whatever failures the elements experience, the friction angle should be kept invariant, and the bulk and shear modulus should be degraded to the same order of magnitude.

#### 4.2.1. Case Study I: Numerical Analysis on the Double-Fissured Specimen under Uniaxial Compression without Fissure Water Pressure

**Figure 7:**
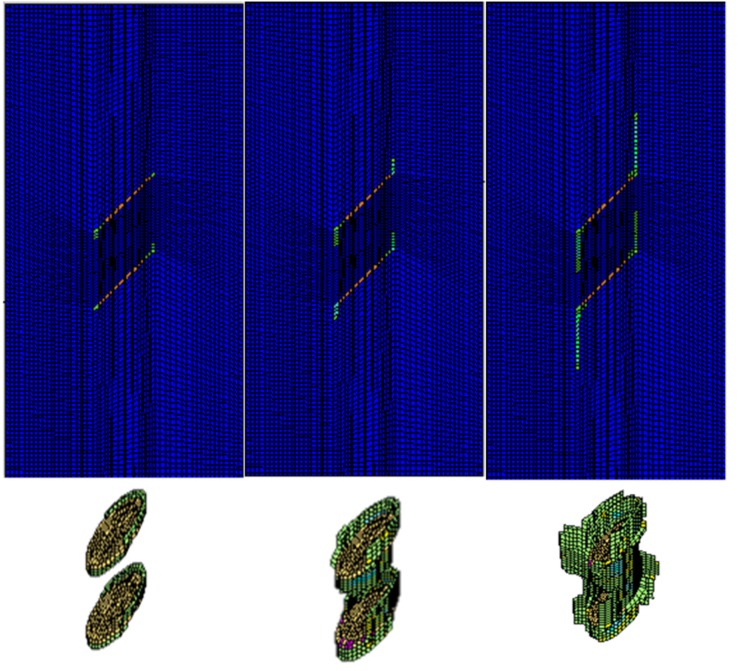
The fissure development and profiles of secondary cracks.

In this case, the double-fissured specimen under uniaxial compression without fissure water pressure is numerically simulated based on the elastic brittle model. Figure 7 shows the fissure development and the profile of the secondary cracks.

During the beginning of uniaxial compressive loading, we could observe that small encapsulated failure planes initiate near the long-axis tips of the elliptical fissure sheet; the secondary cracks start to extend along the loading direction, and the wing cracks are formed. Although the secondary cracks initiate and extend gradually, they have no coalescence areas, and the rock mass between the two fissure sheets remains intact at this stage. As the loading continues to increase to 44.7% of the peak compressive strength, a major failure zone induced by the propagation of secondary cracks is formed in the rock bridge. Afterwards, the failure planes start to extend along the fissure edges until the final failure occurs. The peak compressive strength is 58.2 MPa.

**Figure 8:**
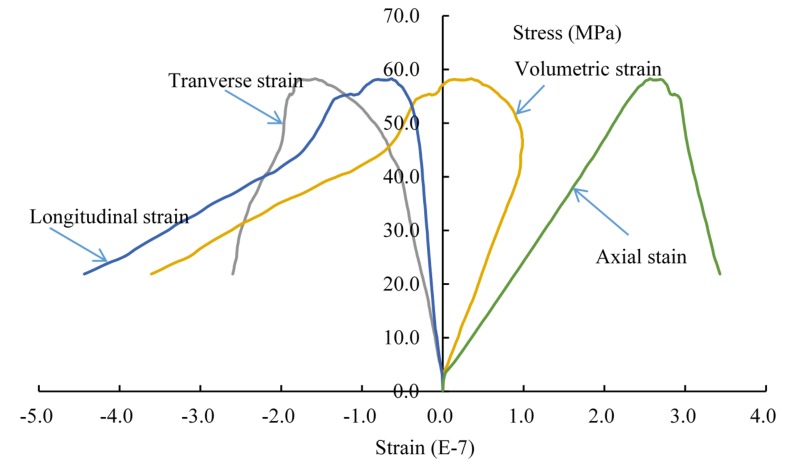
The complete stress-strain curve of the double-fissured specimen under uniaxial compression without fissure water pressure.

The complete stress-strain curve is drawn in Figure 8. In the beginning of loading, the specimen is in the linear elastic stage. When the curves approach the peak values, the axial and transvers strains increase faster, and the specimen appears dilatancy. When the peak strength appears, the stress decreases rapidly. The whole process behaves with the typical characteristics of brittle materials.

#### 4.2.2. Case Study II: Numerical Analysis on the Double-Fissured Specimen under Uniaxial Compression Considering Fissure Water Pressure

**Figure 9:**
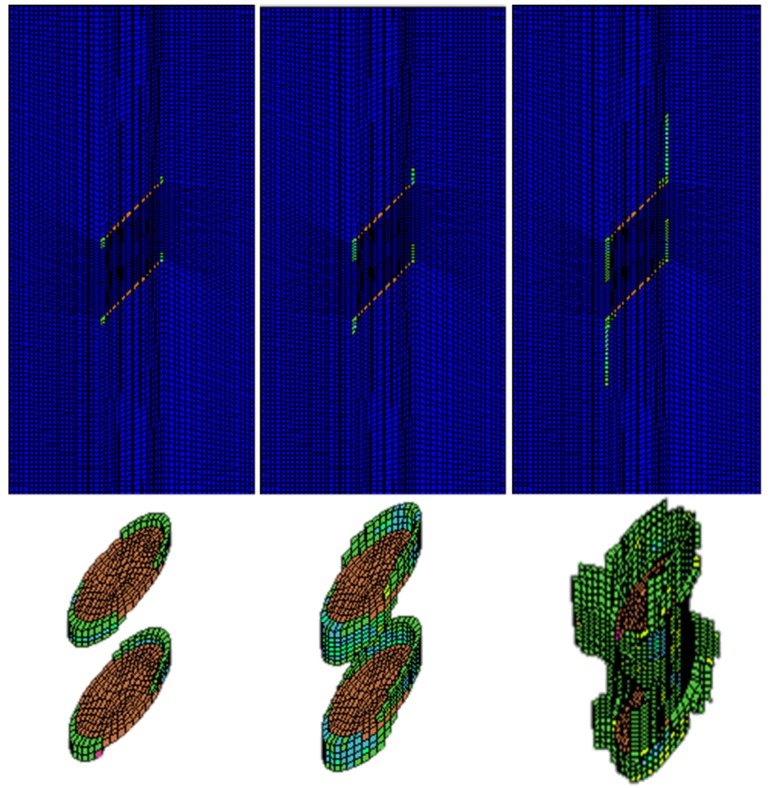
Fissure development and profiles of secondary cracks under fissure water pressure (3.5% *σ_α_*).

**Figure 10:**
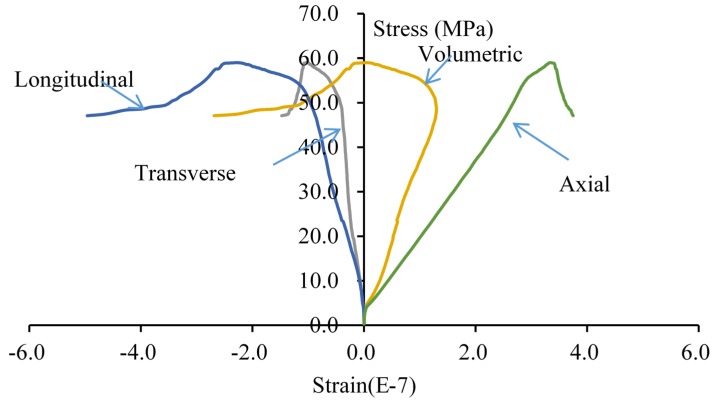
The complete stress-strain curve of the double-fissured specimen under uniaxial compression under fissure water pressure (3.5% *σ_α_*).

**Figure 11:**
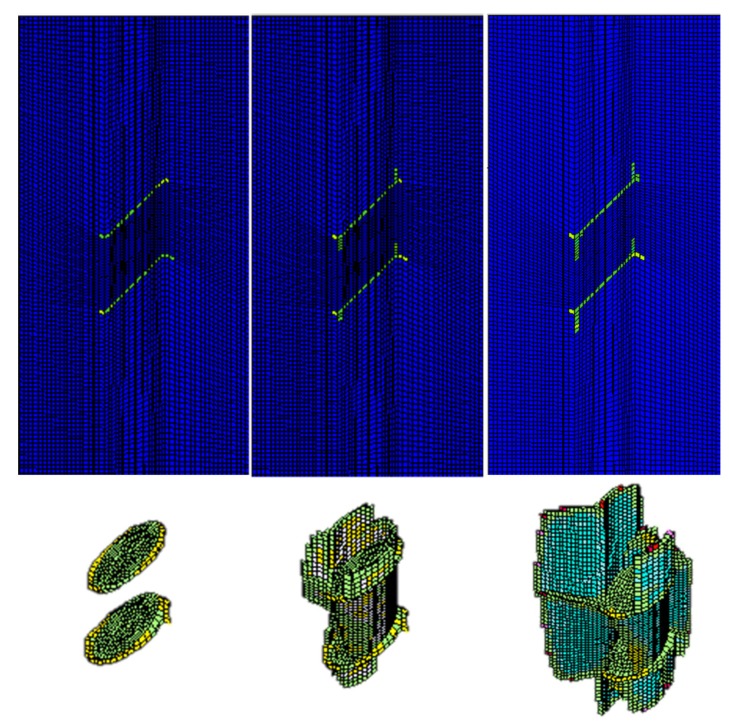
Fissure development and profiles of secondary cracks under fissure water pressure (7% *σ_α_*).

**Figure 12:**
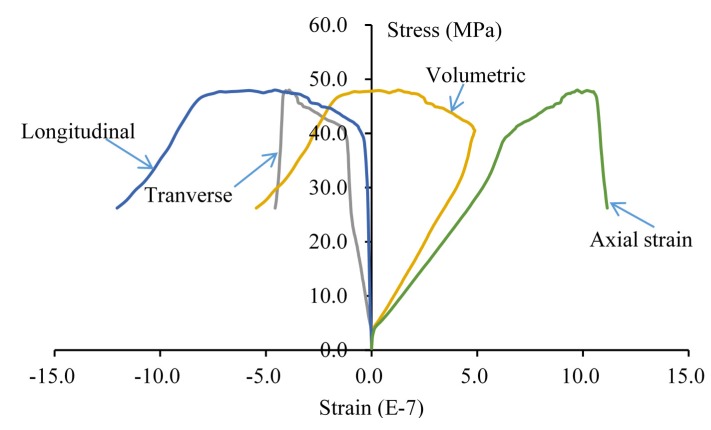
The complete stress-strain curve of the double-fissured specimen under uniaxial compression under fissure water pressure (7% *σ_α_*).

In this case, the double-fissured specimen under uniaxial compression considering fissure water pressures is numerically simulated based on the elastic brittle coupling model. According to the actual laboratory experiments, two representative fissure water pressures are considered. One is 3.5 percent of the uniaxial peak strength (3.5% *σ_α_*), and the other is 3.5% *σ_α_*. Figure 9 shows the fissure development and profiles of secondary cracks under fissure water pressure (3.5% *σ_α_*). Figure 10 is the complete stress-strain curve under fissure water pressure (3.5% *σ_α_*). Figure 11 and Figure 12 show the numerical results under fissure water pressure (7% *σ_α_*).

The following results could be concluded from the numerical simulations:

(1) When the fissure water pressure is equal to 3.5% *σ_α_*, the final uniaxial peak strength is 59.0 MPa. At the beginning of loading, we could find that the fissure development has a similar principle. As the loading continues to increase to 41.3% of the peak compressive strength, a major failure zone induced by the propagation of secondary cracks is formed in the rock bridge. Therefore, it is concluded that the fissure water pressure (3.5% *σ_α_*) has a slight impact on the fissure development.

(2) As the fissure water pressure is increased to 7% *σ_α,_* its impact could not be neglected. The final uniaxial peak strength has obviously been decreased to 42.2 MPa. The cracks initiate and extend while the uniaxial load is 2.11 MPa. As the loading continues to increase, the cracks extend rapidly, because the fissure water pressure intensifies the tensile effects at the fissure tips. When the uniaxial loading is 27.85 MPa, a major failure zone induced by the propagation of secondary cracks is formed in the rock bridge. Afterwards, the failure planes start to extend along the fissure edges until the final failure occurs.

## 5. Conclusions

(1) An elastic brittle coupling constitutive model on the basis of secondary development in FLAC3D is proposed to simulate the fracture development of jointed rock mass under fracture water pressure. The two-dimensional numerical results are found to be in good agreement with the laboratory results.

(2) The fissure water pressure has a significant impact on the peak strength of pre-cracked rock specimen. When the value is small, the peak strength may be increased a little. However, when it increases to a bigger value, the peak strength would be decreased rapidly, and a large-area failure zone would appear in the rock bridge. The failure planes start to extend along the fissure edges until the final failure occurs.
